# Consumers’ Acceptability and Perception of Edible Insects as an Emerging Protein Source

**DOI:** 10.3390/ijerph192315756

**Published:** 2022-11-26

**Authors:** Marta Ros-Baró, Violeida Sánchez-Socarrás, Maria Santos-Pagès, Anna Bach-Faig, Alicia Aguilar-Martínez

**Affiliations:** 1 Faculty of Health Sciences, Open University of Catalonia (UOC), 08018 Barcelona, Spain; 2 FoodLab Research Group, Faculty of Health Sciences, Open University of Catalonia (UOC), 08018 Barcelona, Spain; 3 Unesco Chair on Food, Culture and Development, Open University of Catalonia (UOC), 08018 Barcelona, Spain; 4 Food and Nutrition Area, Barcelona Official College of Pharmacists, 08009 Barcelona, Spain

**Keywords:** edible insects, food preferences, entomophagy, nutrition surveys, food choice, food neophobia

## Abstract

In recent years in Western Europe, studies on entomophagy have drawn the attention of many researchers interested in identifying parameters that could improve the acceptability of insect consumption in order to introduce insects as a sustainable source of protein into the future diet. Analysing the factors involved in consumer acceptability in the Mediterranean area could help to improve their future acceptance. A cross-sectional study was conducted using an ad-hoc questionnaire in which 1034 consumers participated. The questionnaire responses allowed us to study the areas relevant to acceptance: neophobia, social norms, familiarity, experiences of consumption and knowledge of benefits. Only 13.15% of participants had tried insects. Disgust, lack of custom and food safety were the main reasons for avoiding insect consumption. Consequently, preparations with an appetising appearance need to be offered, with flours being the most accepted format. The 40–59-year-old age group was the one most willing to consume them. To introduce edible insects as food in the future, it is important to inform people about their health, environmental and economic benefits because that could increase their willingness to include them in their diet.

## 1. Introduction

The substantial improvements in people’s health status, hygiene conditions and life expectancy, in the majority of countries over the past 50 years, means that the world population is predicted to increase considerably by 2050 [1]. The rising cost of animal protein production and the increasing environmental pressure on agriculture and livestock farming [2] necessitate the search for productive alternatives and innovative techniques for food production which take into account the nutritional, environmental and sociocultural dimensions of food sustainability [3,4]. 

The use of insects as human food could meet these demands and prove to be a valid strategy for improving global food security FAO [5]. Compared to conventional livestock, insect production has a higher conversion rate to food. Insects can grow in organic waste (thereby acting as bioconverters), occupy less production space and produce less greenhouse gases [6,7,8]. For example, when compared to beef production, Ros-Baró et al. [9] found that insect production was responsible for around 95% less greenhouse gas emissions, land and water use, and 62% less energy use. Regarding their nutritional composition, edible insects have bioactive compounds that are beneficial to health: they have the ability to improve intestinal microbiota and are claimed to have not only antioxidant properties, but also to improve some blood parameters [9]. Although nutritional composition depends on the type of insect, its stage of growth and its food, all insects generally have high levels of essential amino acids [10] and highly soluble proteins capable of forming gels or emulsions with a digestibility rate of 78–98%. They contain unsaturated fatty acids [11], micronutrients (riboflavin, pantothenic acid, biotin, thiamine, Vitamin B12, iron, zinc and calcium) and dietary fibre [12,13,14,15]. Despite these advantages, including new foods in a diet is a complex issue that requires consumer acceptance and finding a place for them in the culinary system [16,17]. 

Neophobia, or the refusal—in this case—to try new foods, is one of the main factors influencing the acceptability of edible insects [18,19,20,21]. According to Faccio et al. [22,23], people with food neophobia are also more reluctant to try insects. The degree of rejection is related to dislike or disgust, and to a belief that their consumption is associated with cultures from distant and generally low-income countries [17,24]. The refusal to consume insects is based on cultural reasons, since they are considered unpleasant and, in some cases, harmful, or on doubts about the feasibility and viability of farming them safely [24,25].

Entomophagy was a common practice among our ancestors and has been acknowledged as an important role in human development [26,27]. Numerous references to this practice have been found in the literature and history of the ancient peoples of China and the peoples of the Roman Empire, as well as in the sacred writings of Christianity, Judaism and Islam [27]. In Western countries, entomophagy was abandoned many years ago [28]. 

Providing information about, having positive experiences of, and—at a gastronomical level—incorporating insects into usual recipes together allow their consumption to be endowed with familiarity and proximity [18,29]. Preparations that can make their appearance more appetising will influence their acceptance and, most likely, their consumption as novel foods [30,31,32].

To date, insects have been very much off the menu in Western cuisine. China, Thailand, Japan, Colombia, Mexico, Peru, Brazil and several African nations are the countries with the greatest tradition of insect consumption [33,34]. The most eaten insects include saturniid caterpillars, beetles, ants, termites, crickets, grasshoppers and palm weevil larvae [35]. In Europe, they appear to be better accepted in Austria, Belgium, Holland and France due to their wider introduction into the food industry as a novel food [36]. However, the edible insect industry is progressing rapidly in order to meet the demand for insects as a food ingredient [36] and is also gaining interest in Western countries [37,38], so more studies in different populations are needed to provide information on factors that may favour the acceptance of insects as food for human consumption.

The aim of this study is to explore the opinions of consumers in Mediterranean Europe of insect consumption. Based on questionnaire responses, the study aims to show the differences between the sociodemographic groups surveyed; to identify which type of insect authorised by the European Food Safety Authority (EFSA) is the most consumed, and in what context; to explore the reasons for refusing to consume them; and to identify which presentations are considered to be the most attractive and what factors might influence potential marketing or consumption to improve the acceptability of insect protein as food for humans in the future. 

## 2. Materials and Methods

### 2.1. Study Design

An observational, descriptive, cross-sectional study was conducted to collect data on the consumption of insects and the potential factors influencing their acceptance as a new source of alternative protein in a Spanish population sample. The data collection tool was an ad-hoc questionnaire created from a review of previous studies [17,18,19,20,21]. The survey was prevalidated by researchers from the FoodLab group to assess the relevance and appropriateness of the questions. The process of administering the survey was then piloted with a small sample of known persons. After analysing the responses, changes were made to the initial questionnaire used. The final version consisted of 18 questions relating to the potential factors influencing the acceptance of insect consumption, such as neophobia, social and cultural norms, familiarity, perceived benefits or visual characteristics of the preparation or presentation. Nine questions had a binary Yes/No response option, and nine had multiple options to choose from. The questionnaire also included sociodemographic data, such as the respondents’ gender, age and place of residence.

### 2.2. Participants

The study population comprised adults who mainly resided in Catalonia (Spain) and who voluntarily agreed to answer the questionnaire.

### 2.3. Administration of the Questionnaire

The questionnaire was created on the Qualtrics platform which specialises in online surveys, and was distributed via social media in September 2022. The first screen contained general information about the study. Prior to completing the questionnaire, each participant had to give consent to participate. To ensure the confidentiality of the results obtained, the questionnaires were anonymous and participants could not be identified.

The study was conducted in compliance with the ethical principles for research involving human beings and the processing of personal data contained in the Declaration of Helsinki and was approved by the Ethics Committee of the Open University of Catalonia, CE22-PR28.

### 2.4. Data Analysis

All responses were analysed using the SPSS version 15.0 for Windows. Yes/No responses were considered nominal and dichotomous categorical variables. Pearson’s χ² test, which considers a non-parametric test to measure the differences between an observed distribution and a theoretical one, allowed the relationship between these dichotomous variables to be analysed. In the descriptive analysis of data, demographic characteristics and questions with multiple options were expressed as absolute and relative frequencies. For all calculations, a 95% confidence interval was used and relationships of *p* < 0.005 were deemed statistically significant. The results obtained are shown in descriptive tables for the demographic characteristics of the sample, tables of the relationship between the dichotomous variables and their distribution by the participants’ gender and age group and descriptive tables of the preferred consumption formats or contexts.

## 3. Results

The survey was answered by a total of 1034 participants, of whom 68.85% were women and 66% were over 40 years of age. Table 1 shows the sociodemographic characteristics of the participants by gender, age and province of residence. The participants were mainly distributed between the two most populated provinces of Catalonia (Spain).

While most participants (79.8%) expressed interest in trying new foods or being innovative with their cooking, only 48.2% reported that they had tried new foods in the past year. Of these, quinoa and plant-based foods for vegetarians or vegans were the most widely chosen options. Sushi or soya were also among the most frequently mentioned foods. In terms of insect consumption, 86.9% of participants indicated that they had not consumed them and were unwilling to cook them (71%) or to include them in their usual diet (82.2%). Disgust, followed by lack of custom, and safety concerns were the main reasons given by the participants to justify their lack of interest in consuming insects. However, flour-based preparations were the most attractive option in the event of having to consume them (23.5%), followed by biscuits and bars (around 6%). Of those in “Others” (*N* = 162) and on which information was available, the most preferred options, in descending order, were the following: powders, flakes, sweets, burgers and meatballs. Among those who stated that they had eaten insects, the most consumed one was the cricket (5.2%), followed by mealworms (4.8%). Table 2 shows the distribution of participants’ responses to the questionnaire.

Analysis of the responses by gender showed significant differences (*p* < 0.001) between men and women regarding their willingness to consume insects, with women being less willing to cook and include them in their diet in a usual manner (Supplementary material, Supplementary Table S1). Likewise, men were more willing than women to consume them in preparations where the whole insect could be seen.

Significant differences were observed in insect consumption by age (Supplementary material, Supplementary Table S2), with those over 60 years of age reporting lower consumption of insects and less intention to consume them. Similarly, they were less willing to try new foods. The age group that was most familiar with insect consumption was the 40–59-year-old one, at 7.2%. These results contrast with the perception, expressed by respondents, of a greater acceptance by adolescents. They considered adolescents to be the age group that would be the most willing to welcome insect consumption, and older adults to be the one least willing to do so. 

The context or circumstances in which insects were introduced proved to be different from that of the consumption of other foods. 

**Figure 1:**
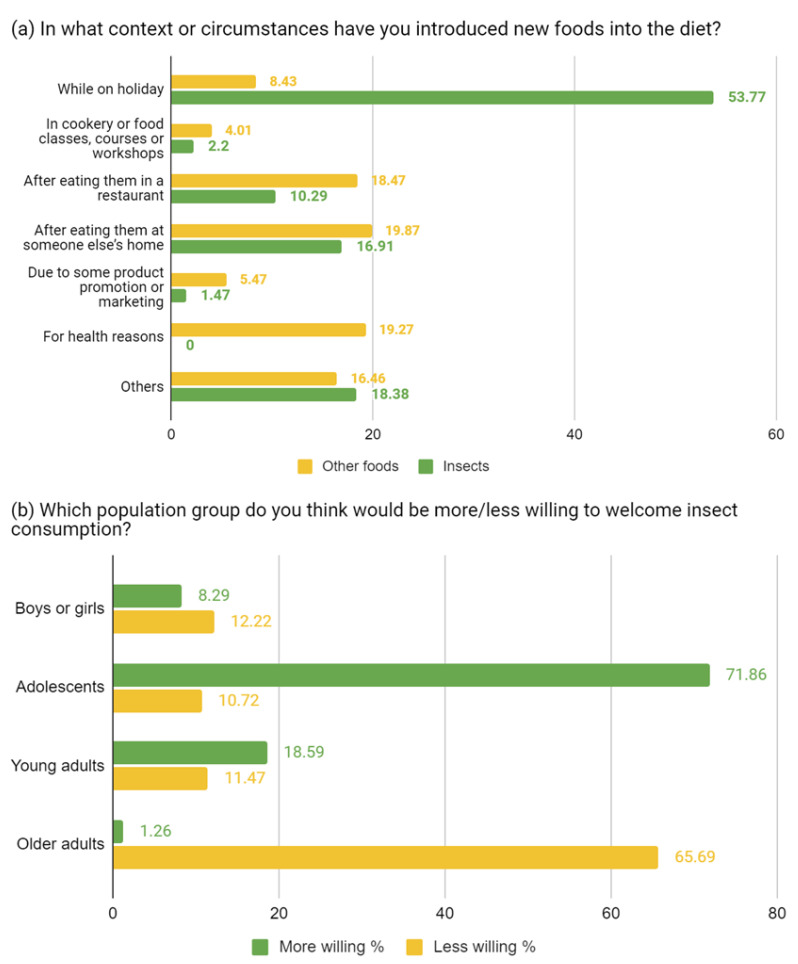
Distribution of food introduction contexts and level of reception by population group. (**a**) Context or circumstances of introduction of new foods into the diet. (**b**) Population group and willingness to welcome insect consumption.

When the respondents were asked in what context or circumstances they usually introduced new foods into their usual diet, differences regarding insect consumption were found. Participants who reported having consumed insects (13.15%) had done so mostly while on holiday (53.77%). Regarding the other foods, most acknowledged that they had introduced them after consuming them at someone else’s home (19.87%) or for health reasons (19.27%) (Figure 1). Although the response to the general question about whether insect-based dishes would be welcomed by the general public was negative, the possibility of offering them in restaurants was more plausible since it decreased negativity by 3.4% compared to the previous question. 

Although the majority of study participants would not include insects in their usual diet (82.2%), they were much more positive about their future incorporation (58.3% considered insect eating to possibly be a usual practice in the future). Furthermore, knowing their potential benefits for sustainability improved their willingness to consume them. As the results in Table 3 show, when relating respondents’ willingness to include insects in their usual diet, it was found that while 51.9% responded that they would not try them, 56.17% would do so because they were a sustainable protein. The relationship between dichotomous variables was statistically significant (*p* = 0.001). 

## 4. Discussion

This study investigated consumer perception of the inclusion of edible insects in human food and showed their poor acceptance by the studied population. Consistent with the studies by [17,18], neophobia was found to be a key obstacle to the acceptance of such products, despite the fact that more and more people appear to be willing to incorporate new foods into the food pattern of the Mediterranean diet, which is typical of the study population [38]. The respondents’ mentions of quinoa, sushi or soya as foods recently introduced into the diet reflect the effects of market globalisation on food. Similarly, other recently incorporated products were foods for vegetarians and vegans (10.8%), which is consistent with the observed increase in this trend in society [39,40]. Insect consumption was low, as only 13.1% of respondents mentioned that they had consumed them. This is a higher percentage than that obtained in previous studies, such as the one by Verbeke et al. [17].

Some previous studies have suggested that young people may be more attracted to insect consumption and, for that reason, their degree of neophobia of trying new foods is lower than other age groups, such as young adults or older adults Verbeke et al. [17]. However, contrary to the findings of Verbeke et al. [17], this study found that the 40–49-year-old group was more willing to accept insect consumption, unlike the findings of Hartman et al. [20] where age was not associated with willingness to eat insects. 

Consistent with other studies [18], male respondents in this study appeared to have a lower degree of neophobia and were more willing to cook insects and to introduce them into the usual diet than female respondents were. 

According to the results obtained from the survey, insects were mostly consumed during a trip to countries where there was a tradition of eating them. While such an experience may be an initial opportunity to try the product and then to incorporate it if the experience is positive [41], in many cases the first experience is of preparations that include whole insects, and this may give rise to even more neophobia among the Western population [42,43]. In addition, there is a marked difference between eating raw and cooked insects, and the incorporation of other ingredients and cooking processes can improve their acceptance at multiple levels [44]. The results of this study show that 69.8% of participants would prefer preparations where the natural appearance could not be seen, which is consistent with other studies [3,20,45,46,47] which assert that consumers in Western cultures are more willing to eat a processed product than a whole one. Besides the visual characteristics, the willingness to consume new products is favoured by a familiarity with them [20]. In this sense, flour was the preferred format for respondents, so its incorporation into foods such as bread or biscuits may determine better acceptability and a lower degree of neophobia given their familiarity to the Mediterranean population. In addition, it has been reported that personal participation in culinary preparations reduces the degree of disgust felt [8,48]. The use of insect flour as an ingredient would be easy to incorporate into multiple recipes of the cultures of Mediterranean Europe [49,50]. In the quest for strategies or presentations that disguise the presence or shape of insects to meet consumer demand [51], the food industry has identified their potential use in seasoning powders for soups instead of commercial products made from pork or chicken, margarines, milk or burgers [52,53,54,55] and also as an emulsifier [45]. Strategies introducing the partial replacement of meat with sustainable protein sources, such as vegetables and insect flours, were successfully employed in food product formulations containing less animal protein [56]. Likewise, Spence et al. [52] have described the application of techniques used in haute cuisine.

Social and cultural norms are also factors that determine food customs and the incorporation of foods [53]. Social acceptance is a significant predictor of the willingness to eat insects, since entomophagy is deemed a primitive practice [25,48] or a source of nutrients in times of economic scarcity [57]. In this study, however, neither the consideration of insect eating as a primitive practice nor the relationship to low economic resources appeared to be important barriers to consumption. While insect preparations might be considered delicacies in Western countries, they are considered a food for basic use, or for use during food emergencies, in other parts of the world [58]. The lack of custom or doubts about insect safety seem to have the greatest impact on the food choice and, after disgust, they are the main reasons for rejection. There are also concerns about the possible presence of pathogenic organisms and heavy metals, and about the potential allergic reactions to their consumption [8,37,59,60]. At every stage of edible insect processing (from farm to fork), control measures and hazard analysis and critical control points (HACCPs) are needed to reduce the risk of foodborne propagation [44,60,61]. In this sense, the positive opinion issued in 2021 by the EFSA—a trusted institution for Western societies—on the safety of the mealworm (*Tenebrio molitor larva*) [62], the migratory locust (*Locusta migratoria*) [63] and the cricket (*Acheta domesticus*) [64] as novel foods (Roma 2020) under Regulation (EU) 2015/2283 [65] could help to dispel doubts about the potential risk to human safety [66] and contribute to a greater willingness to consume them [21]. Globally, there are few legal instruments that treat insects as food [67], so greater sensitisation and awareness-raising would be needed to inform people about the benefits and safety of authorised insects if the aim is to introduce them into the diets of populations that do not have a tradition of entomophagy. 

The nutritional benefits of insects and their value as a more sustainable source of protein are of great interest to Western society [12]. These benefits may be of particular interest to groups where protein needs are greater due to their life situation (older adults, athletes, etc.) [13], to societies where protein alternatives are sought due to the scarcity of traditional ones [8] or to raise awareness of the environmental impact of alternative sources due to the risk of surpassing planetary limits [68,69]. In any case, to take advantage of the benefits of insects as an alternative protein source, the proportion of insect proteins included in products must be comparable to that of other common protein sources [9].

Information on the benefits associated with insect consumption influences their acceptance [70]. The results of our study confirm a greater willingness to consume insects when people are informed of the potential sustainability benefits of doing so, increasing the possible acceptability thereof by 36.3%. The connection with the sustainability and well-being of the planet is a social trend that could favour the introduction of insects into the diet [1]. Insect farming for human consumption appears to offer several environmental benefits [71]. These include the use of organic waste, its added value and the reduction of environmental pollution. In addition, it leads to a reduction in greenhouse gas emissions [72], lower water consumption and higher food conversion efficiency [7]. 

While the study does not mention any potential health benefits of insect consumption, health was one of the most common reasons given for including other novel foods by those who had already done so. This suggests that informing people of the potential health benefits of insect consumption could also improve the willingness to consume insects, as is the case with sustainability. 

The implementation of novel, sustainable food production strategies, as is the case with insects, may help to meet several United Nations sustainable development goals as defined by Moruzzo et al. [73]. However, the marketing and consumption of insects as food must strike a balance between regulation, environmental impact, social and market demands and public health needs and prospects [74]. Likewise, culinary preparation procedures and techniques adapted to the sociocultural context must be developed [75]. The market for edible insects is an emerging economic sector supported by academic research and innovation in the private sector (from processing to selling) [21], and it is an easily accessible and economical product [76]. Nevertheless, to boost future lines of production, more pilot tests of acceptability are needed with products that are more familiar to Western society, and more positive experiences need to be generated. 

One of the potential limitations of this study is that the convenience sampling method used may have led to a bias relating to the participation of people who were more interested in or motivated by the subject, or who had a higher educational level. Another aspect to consider is that the majority of the responses belonged to the binary Yes/No option. No acceptance scales were used and no account was taken of whether respondents were following any kind of diet. Finally, the survey uses the term insect, which evokes an association with visible and whole insects [77], so perception may have influenced response. Despite these limitations, the study provides valuable information on the main factors that could improve the acceptability of insect consumption in order to introduce insects as a sustainable source of protein into the future diet. The data were drawn from the responses given by 1034 participants, a large number that exceeds other studies on the perception of insects as food in Mediterranean countries. Likewise, conducting a survey in a Mediterranean environment allows a broader view of Western consumer opinion, unlike previous surveys [15,16,17,18,19] whose focus was on consumer opinions in Northern and Central Europe.

## 5. Conclusions

In the near future, edible insects may appear in Western food in response to the need to look for new and more sustainable sources of alternative protein within the framework of sustainable development goals. Our data corroborate the low consumer acceptance of the inclusion of edible insects in human food through areas relevant to acceptance: neophobia, social norms, familiarity, experiences of consumption and knowledge of benefits. Disgust, lack of custom and food safety are the main reasons for neophobia. Neophobia has previously been studied in other populations, but not in a large sample of the Mediterranean population until now. At the gender level, men are more willing to consume insects, and so too are those in the 40–59-year-old age group.

The environmental and nutritional benefits of this type of product can open the door to the consumption of this novel food, which has been accepted by the EFSA in Europe. Informing people of such benefits for the health of the planet can improve their perception of insects and encourage them to consume them. However, the need to go further and offer products that make edible insects more familiar to Western society is identified. Producing commonly used flour-based products (bread, biscuits, bars, etc.) and offering culinary preparations closer to regional culture are ways to do that.

## Figures and Tables

**Table 1:** Sample characteristics.

	*N*	%
Gender		
Female	712	68.86
Male	321	31.04
Non-binary	1	0.1
Age		
18–24	160	15.47
25–39	191	18.47
40–49	274	26.5
50–59	341	32.98
60 or over	68	6.58
Resident in Catalonia		
Tarragona	498	48.16
Barcelona	420	40.62
Girona	50	4.83
Lleida	24	2.32
Resident outside Catalonia	42	4.06

**Table 2:** Distribution of participants’ responses to the questionnaire.

	*N*	%
When it comes to cooking, do you like trying new things or being innovative with how you prepare your food?		
Yes	825	79.78
No	209	20.22
In the past year, have you introduced new foods into your diet?		
Yes	498	48.16
No	536	51.84
If so, select the foods introduced into the diet		
Tropical fruits	50	4.83
Kefir	79	7.64
Tofu	56	5.41
Seaweed	61	5.89
Sushi	94	9.09
Quinoa	143	13.82
Oats	98	9.47
Soya	74	7.15
Shiitake	43	4.15
Foods for vegetarians or vegans	112	10.83
Others	135	13.05
Have you ever eaten insects?		
Yes	136	13.15
No	898	86.85
If so, what insects have you eaten?		
Crickets	54	5.22
Grasshoppers	39	3.77
Mealworms	50	4.83
Others	39	3.77
Main reasons for not consuming insects		
Disgust	395	38.2
Doubts about safety	98	9.47
It seems to me to be a primitive practice	3	0.29
It seems to me to be an option only for societies with few economic resources	6	0.58
Lack of knowledge	17	1.64
Lack of custom	159	15.37
Cultural reasons	68	6.57
Others	118	11.41
Would you include insects in your usual diet?		
Yes	171	16.54
No	850	82.2
No response	13	1.26
Would you be willing to cook insects at home?		
Yes	290	28.05
No	735	71.08
No response	9	0.87
Would you offer insect-based dishes in a restaurant?		
Yes	259	25.04
No	764	73.89
No response	11	1.06
Do you think insect-based dishes would be welcomed by the general public?		
Yes	170	16.44
No	846	81.82
No response	18	1.74
Would knowing that insect consumption has the potential to be a sustainable food practice encourage you to consume them?		
Yes	511	49.42
No	499	48.25
No response	24	2.33
Do you think insect consumption might become a common practice in the future?		
Yes	603	58.32
No	403	38.97
No response	28	2.71
In what preparations do you think insects would be more attractive?		
If their natural appearance cannot be seen	722	69.82
If their natural appearance can be seen	102	9.87
No response	210	20.31
Which presentations do you find more attractive?		
Flours	243	23.5
Bars	60	5.8
Gels	1	0.09
Jellies	5	0.48
Biscuits	63	6.09
Pills	24	2.32
Smoothies	23	2.22
Others	162	15.66

**Table 3:** Relationship between willingness to include insects in the usual diet and willingness to include them knowing that doing so has the potential to be a sustainable practice.

Insects Usual Diet	Contribution to SustainabilityNo *N* (%)	Contribution to Sustainability Yes *N* (%)	Total
No	301 (60.32)	224 (43.83)	525 (51.98)
Yes	198 (39.68)	287 (56.17)	485 (48.02)
Total	499 (100)	511 (100)	1010 (100)

Pearson’s Chi^2^ (1) = 26.3751 *p* = 0.0013. Excludes no response to each item

## Supplementary Materials

The following supporting information can be downloaded at: https://www.mdpi.com/article/10.3390/ijerph192315756/s1, Table S1: Distribution of questionnaire responses by the participants’ age (95% CI); Table S2: Distribution of questionnaire Distribution of questionnaire responses by the participants’ gender (95% CI) responses by the participants’ age (95% CI).
