# Supplementary material for: Consumers’ Acceptability and Perception of Edible Insects as an Emerging Protein Source
Source: Int J Environ Res Public Health. 2022 Nov 26;19(23):15756. doi: 10.3390/ijerph192315756 (PMC9739510; doi:10.3390/ijerph192315756)
Supplement: Supplementary file 1 [file ijerph-19-15756-s001.zip › Table S1.pdf]

Table S1. Distribution of questionnaire responses by the participants' gender (95% CI).

	Male N (%)	Female N (%)	B (95% CI)
**When it comes to cooking, do you like trying new things or being innovative with how you prepare your food?**			p=0.632
Yes	258 (24.95%)	566 (54.73%)	
No	62 (5.99%)	147 (14.21%)	
**In the past year, have you introduced new foods into your diet?**			p=0.568
Yes	148 (14.31%)	350 (33.84%)	
No	172 (16.63%)	364 (35.2%)	
**Have you ever eaten insects?**			p=0.008
Yes	50 (4.83%)	86 (8.31%)	
No	270 (26.11%)	628 (60.73%)	
**Would you include insects in your usual diet?**			p<.001
Yes	84 (8.12%)	87 (8.41%)	
No	233 (22.53%)	617 (56.67%)	
**Would you be willing to cook insects at home?**			p<.001
Yes	139 (13.44%)	151 (14.6%)	
No	180 (17.4%)	555 (53.67%)	
**Would you offer insect-based dishes in a restaurant?**			p=0.058
Yes	112 (10.83%)	147 (14.21%)	
No	204 (19.72%)	560 (54.15%)	
**Do you think insect-based dishes would be welcomed by the general public?**			p=0.037
Yes	64 (6.18%)	106 (10.25%)	
No	251 (24.27%)	595 (57.54%)	
**Would knowing that insect consumption has the potential to be a sustainable food practice encourage you to consume them?**			p=0.007
Yes	185 (17.89%)	326 (31.52%)	
No	127 (12.28%)	372 (35.97%)	
**Do you think insect consumption might become a common practice in the future?**			p=0.324
Yes	212 (20.5%)	391 (37.81%)	
No	100 (9.67%)	303 (29.3%)	
**In what preparations do you think insects would be more attractive?**			p=0.003
If their natural appearance cannot be seen	186 (17.98%)	536 (51.83%)	
If their natural appearance can be seen	47 (4.54%)	55 (5.31%)	

The table excludes the non-binary gender response as there was only one.
