# Supplementary material for: Consumers’ Acceptability and Perception of Edible Insects as an Emerging Protein Source
Source: Int J Environ Res Public Health. 2022 Nov 26;19(23):15756. doi: 10.3390/ijerph192315756 (PMC9739510; doi:10.3390/ijerph192315756)
Supplement: Supplementary file 1 [file ijerph-19-15756-s001.zip › Table S2.pdf]

Table S2. Distribution of questionnaire responses by the participants' age (95% CI).

	18-24 years N (%)	25-39 years N (%)	40-49 years N (%)	50-59 years N (%)	60 years or over N (%)	B (95% CI)
**When it comes to cooking, do you like trying new things or being innovative with how you prepare your food?**						p<.001
Yes	129 (12.47%)	167 (16.15%)	227 (21.95%)	253 (24.46%)	49 (4.73%)	
No	31 (2.99%)	24 (2.32%)	47 (4.54%)	88 (8.51%)	19 (1.83%)	
**In the past year, have you introduced new foods into your diet?**						p<.001
Yes	57 (5.51%)	100 (9.67%)	153 (14.79%)	150 (14.5%)	38 (3.67%)	
No	103 (9.96%)	91 (8.8%)	121 (11.7%)	191 (18.47%)	30 (2.9%)	
**Have you ever eaten insects?**						p<.001
Yes	18 (1.74%)	32 (3.09%)	35 (3.38%)	39 (3.77%)	12 (1.16%)	
No	142 (13.73%)	159 (15.37%)	239 (23.11%)	302 (29.2%)	56 (5.41%)	
**Would you include insects in your usual diet?**						p<.001
Yes	27 (2.61%)	32 (3.09%)	53 (5.12%)	48 (4.64%)	11 (1.06%)	
No	132 (12.76%)	154 (14.89%)	217 (20.98%)	291 (28.14%)	56 (5.41%)	
**Would you be willing to cook insects at home?**						p<.001
Yes	63 (6.09%)	56 (5.41%)	86 (8.31%)	69 (6.67%)	16 (1.54%)	
No	96 (9.28%)	132 (12.76%)	186 (17.98%)	269 (26.01%)	52 (5.02%)	
**Would you offer insect-based dishes in a restaurant?**						p<.001
Yes	44 (4.25%)	44 (4.25%)	82 (7.93%)	76 (7.35%)	13 (1.25%)	
No	115 (11.12%)	143 (13.82%)	190 (18.37%)	262 (25.33%)	54 (5.22%)	
**Do you think insect-based dishes would be welcomed by the general public?**						p<.001
Yes	27 (2.61%)	30 (2.9%)	54 (5.22%)	49 (4.73%)	10 (0.96%)	
No	132 (12.76%)	157 (15.18%)	215 (20.79%)	286 (27.65%)	26 (5.41%)	

**Would knowing that insect consumption has the potential to be a sustainable food practice encourage you to consume them?**						p=0.071
Yes	92 (8.89%)	100 (9.67%)	142 (13.73%)	140 (13.53%)	37 (3.57%)	
No	63 (6.09%)	86 (8.31%)	128 (12.37%)	193 (12.37%)	29 (2.8%)	
**Do you think insect consumption might become a common practice in the future?**						p=0.071
Yes	98 (9.47%)	116 (11.2%)	170 (16.44%)	174 (16.82%)	45 (4.35%)	
No	58 (5.6%)	72 (6.96%)	98 (9.47%)	157 (15.18%)	18 (1.74%)	
**In what preparations do you think insects would be more attractive?**						p<.001
If their natural appearance cannot be seen	91 (8.8%)	132 (12.76%)	193 (18.66%)	253 (24.46%)	53 (5.12%)	
If their natural appearance can be seen	22 (2.12%)	19 (1.83%)	31 (2.99%)	23 (2.22%)	7 (0.67%)	
